# Treatment of rare neurologic diseases a more and more pressing concern

**DOI:** 10.25122/jml-2018-1007

**Published:** 2018

**Authors:** Victor Lorin Purcarea

In the past decades, diagnosis and treatment of rare diseases has been and still is a priority of scientific research in the health care field. The high prevalence of these diseases and the very high typological diversity has affected millions of people in the whole world. The specialists in the field have identified over 30 million cases only in the European Union and over a million cases in our country, and, unfortunately, for most of them, they were not able to identify the appropriate treatment. This does not mean that the specialists in the field have given up, but it translates into an intensification of conjoined efforts. As rare neurological diseases (**RND**) have a significant prevalence among rare diseases, the European Academy of Neurology (**EAN**) has diversified its efforts to provide patients with a better access to high-quality and safe care, increased European cooperation in the field of high-quality healthcare, exchanges of positive experiences, in order to improve the diagnosis and treatment needed, and prompt dissemination of research results, by creating centers and thus contributing to the development, evaluation and popularization of medical research.

In this context, in 2017, the European Academy of Neurology (**EAN**) together with “Iuliu Hatieganu” University of Medicine and Pharmacy in Cluj-Napoca, RoNeuro Institute for Neurological Research and Diagnosis, Society for the Study of Neuroprotection and Neuroplasticity, Romanian Society of Neurology and the Academy of Medical Sciences organized the **First International Teaching Course for Rare Neurologic Diseases**.

**Figure 1:**
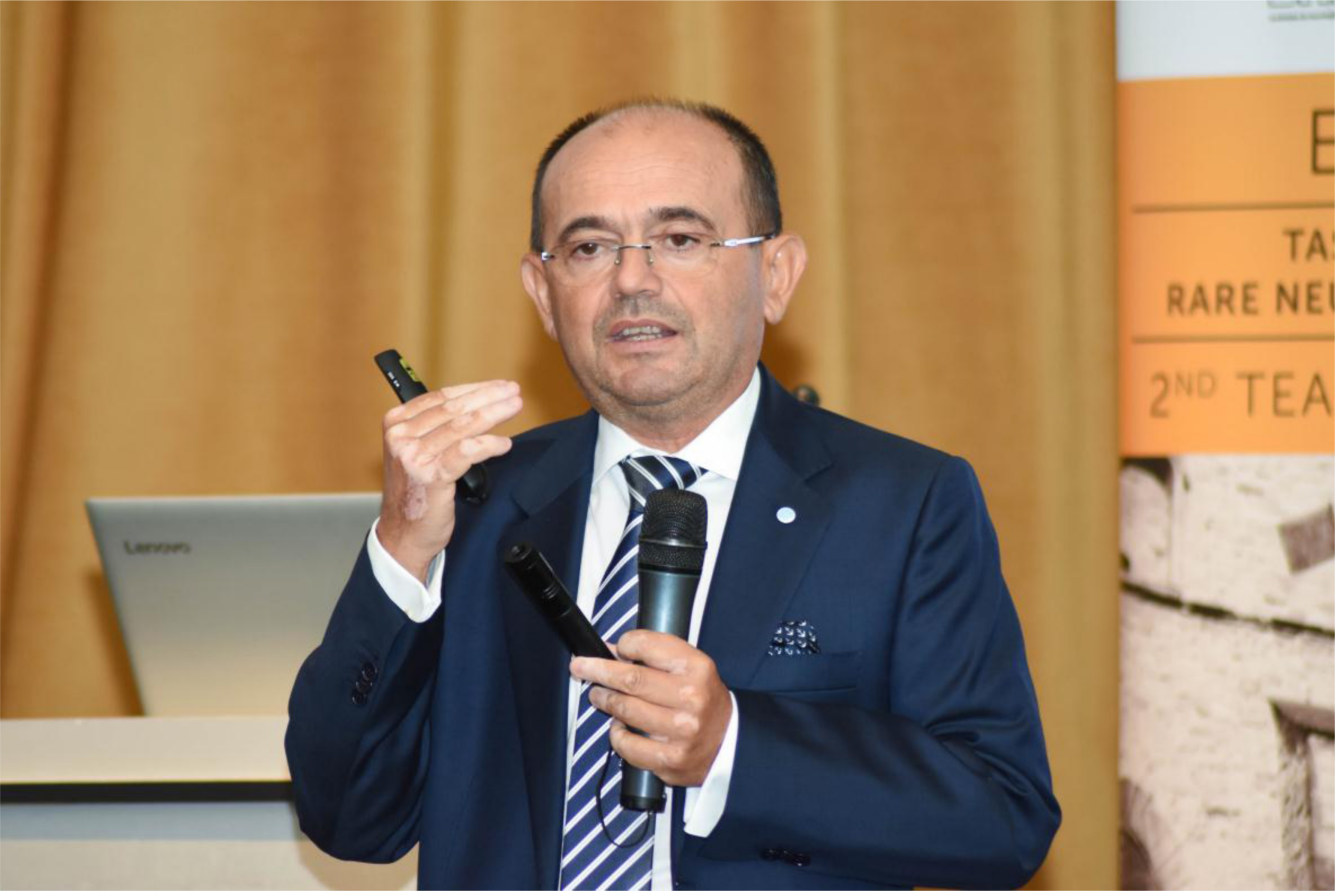
Dafin Fior Muresanu, Chair of EAN Communication and Liaison Committee, Co-Chair EAN Scientific Panel Neurorehabilitation, President European Federation of NeuroRehabilitation Societies (EFNR), Professor of Neurology, Chairman Department of Neurosciences “Iuliu Hatieganu” University of Medicine and Pharmacy, Cluj-Napoca, Romania, Chairman “RoNeuro” Institute for Neurological Research and Diagnostic, and President of the Society for the Study of Neuroprotection and Neuroplasticity (SSNN)

At the end of last year, as a natural consequence of the outstanding achievement of the First edition of the European Academy of Neurology (**EAN**) Task Force meeting, dedicated to rare neurologic diseases, the second meeting took place in Cluj Napoca, at the initiative and with the concrete and effective support of Prof. Dafin Fior Muresanu.

Particularly well received and appreciated, the 2^nd^ European Academy of Neurology (**EAN**) Task Force meeting and the **Rare Neurologic Diseases International Teaching Course** has been an ideal framework for discussion, presentation of the efforts made in the diagnosis and treatment of rare neurologic diseases, highlighting the results of individual research, and identifying new approaches.

Unfortunately, in neurology and not only, malignancy has an undesirably high frequency, and, although great progress has been made, the correct identification of the causes and the establishment of a proper diagnosis and appropriate treatment require extremely long periods of time and the prospect does not seem sure yet.

Prestigious representatives in the European neurology field from Switzerland, Great Britain, Germany, Italy, Belgium, Holland, Finland, Hungary and Romania have participated and the event took place in the generous halls of Grand Hotel Italia, in Cluj-Napoca, generating a wide debate on the evolution of rare neurologic diseases, their diagnosis and treatment, the discovery of new such conditions considered rare, but also the development of special patient records.

**Figure 2:**
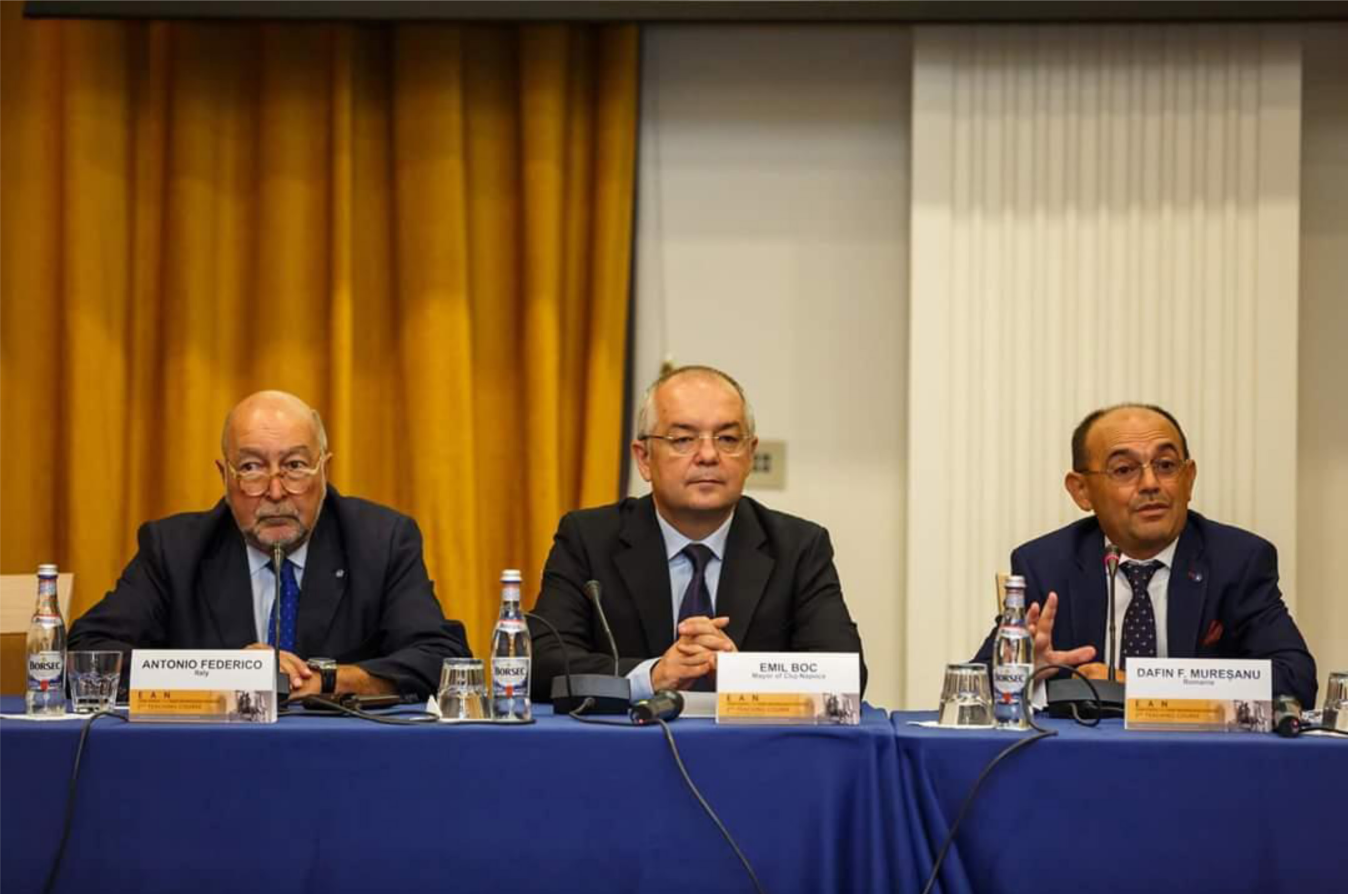
Presidium (from left to right): **Antonio Federico**, Chairman of the Scientific Committee and Member of the Board of the European Academy of Neurology; **Emil Boc**, General Mayor of Cluj Napoca; **Dafin Fior Muresanu**, Chair of EAN Communication and Liaison Committee, Co-Chair EAN Scientific Panel Neurorehabilitation

The event was opened by his Excellency, Emil Boc, General Mayor of Cluj Napoca, and consisted of high interest subjects: Diagnosis and specific care of patients with rare neurologic diseases and the role of the European Academy of Neurology (**EAN**); Position of rare neurologic diseases and of rare neuromuscular diseases (RNM) in the European Reference Network, small vessels disease (SVD), Huntington’s chorea and Chorea-Acathocytosis, limb-girdle muscular dystrophy, inherited peripheral neuropathies, metabolic neuropathies, epilepsy, FSH, spastic paraparesis, dystonias, Mineral and Metal accumulation into the brain, Late onset Metabolic leukoencephalopathies, Optic neuropathies and CTX, the diagnosis improvement methods in rare neurologic diseases, etc.

The meeting also included a round table on current therapeutic opportunities for rare neurologic diseases, attended by all speakers, coordinated by **Antonio Federico**, Chairman of the Scientific Committee and Member of the Board of the European Academy of Neurology and Vice-Rector of the University of Siena, Italy, and **Dafin Fior Muresanu**, Chair of EAN Communication and Liaison Committee, Co-Chair EAN Scientific Panel Neurorehabilitation, President European Federation of NeuroRehabilitation Societies (EFNR), Professor of Neurology, Chairman Department of Neurosciences “Iuliu Hatieganu” University of Medicine and Pharmacy, Cluj-Napoca, Romania, Chairman “RoNeuro” Institute for Neurological Research and Diagnostic, and President of the Society for the Study of Neuroprotection and Neuroplasticity (SSNN).

**Figure 3:**
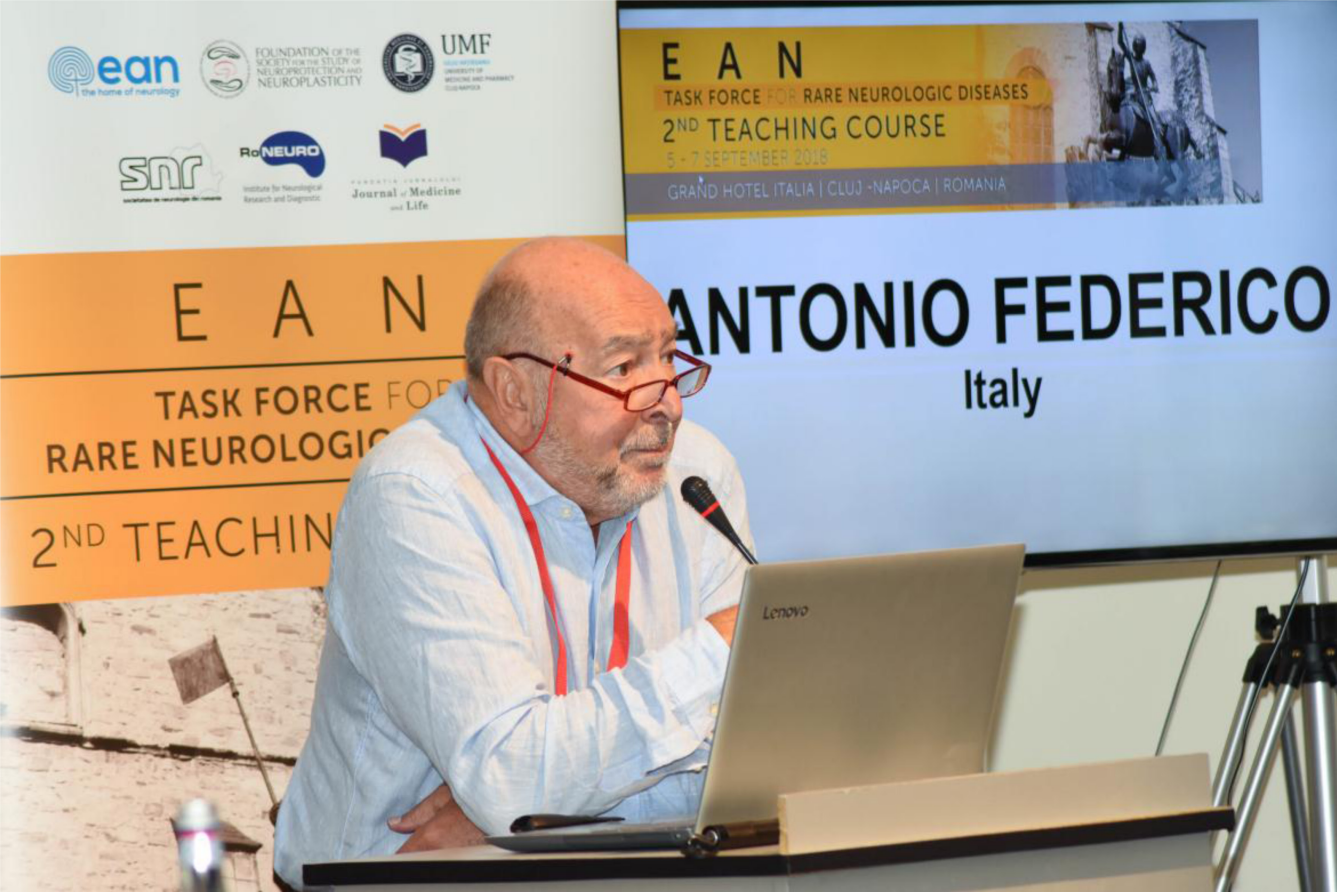
Antonio Federico, Chairman of the Scientific Committee and Member of the Board of the European Academy of Neurology and Vice-Rector of the University of Siena, Italy

The efforts of the European Academy of Neurology (**EAN**) are perfectly justified given that rare neurologic diseases are often underdiagnosed and do not always benefit from efficient treatment. They integrate in the overall efforts to raise awareness and continually improve knowledge in the field, to identify an early diagnosis and special research programs that lead to useful therapies.

The European Academy of Neurology (EAN) joins global collaborative efforts to develop the “so-called orphan drugs”, although in the health industry field, has had many notable successes and recent findings in the genetic fields and biotechnology have forced researchers understand and treat diseases at the molecular level. Information technology is now increasingly included in all health fields and, as stated before, it is necessary that data from different sources, such as laboratory tests, genetic tests, medical images, family history and the patient’s medical record, are easy to store, integrate and analyze.

The increasing convergence of medical science with information technology generates significant research results with an impact on life, but, although there are promising scientific and medical technologies, which can be applied, there are still major implementation difficulties. That is why the efforts of the European Academy of Neurology (**EAN**) have resulted in the joint message sent, highlighting the necessity that the results of the research and the accumulated knowledge should be properly shared and integrated in the context of connecting patients, healthcare providers and health institutions, quickly and efficiently, in the interest of the sole beneficiary: the **patient**.

